# Preparation of nucleoside analogues: opportunities for innovation at the interface of synthetic chemistry and biocatalysis

**DOI:** 10.1039/d5sc03026a

**Published:** 2025-06-05

**Authors:** Admir Salihovic, Andrea Taladriz-Sender, Glenn A. Burley

**Affiliations:** a Department of Pure & Applied Chemistry, University of Strathclyde 295 Cathedral Street Glasgow G1 1XL UK glenn.burley@strath.ac.uk; b Strathclyde Centre for Molecular Bioscience, University of Strathclyde UK

## Abstract

Nucleoside analogues are used throughout nature. They comprise the key building blocks in nucleic acids and in second messenger small molecules. Additionally, modifications to the sugar and nucleobase moieties have been a pervasive feature in the development of nucleoside therapeutics. Despite their ubiquity across all facets of medicinal chemistry and biology, methods to prepare nucleoside analogues are challenging. Recent innovations in the chemical and biocatalytic syntheses of nucleosides offer new opportunities for the step-efficient and environmentally sustainable preparation of these high value analogues. This perspective outlines the key innovations between 2020–2025 and presents opportunities for further integration of these strategies to prepare nucleoside analogues not possible using each of these approaches in isolation.

## Abstract 2

This perspective details contemporary approaches used to prepare nucleoside analogues, and opportunities to integrate aspects of chemical synthesis and biocatalysis to access novel nucleoside chemical space.

## Graphical abstract

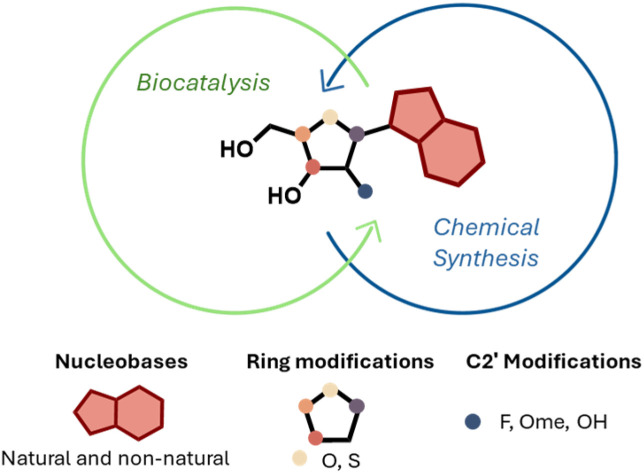

## Introduction

**Fig. 1:**
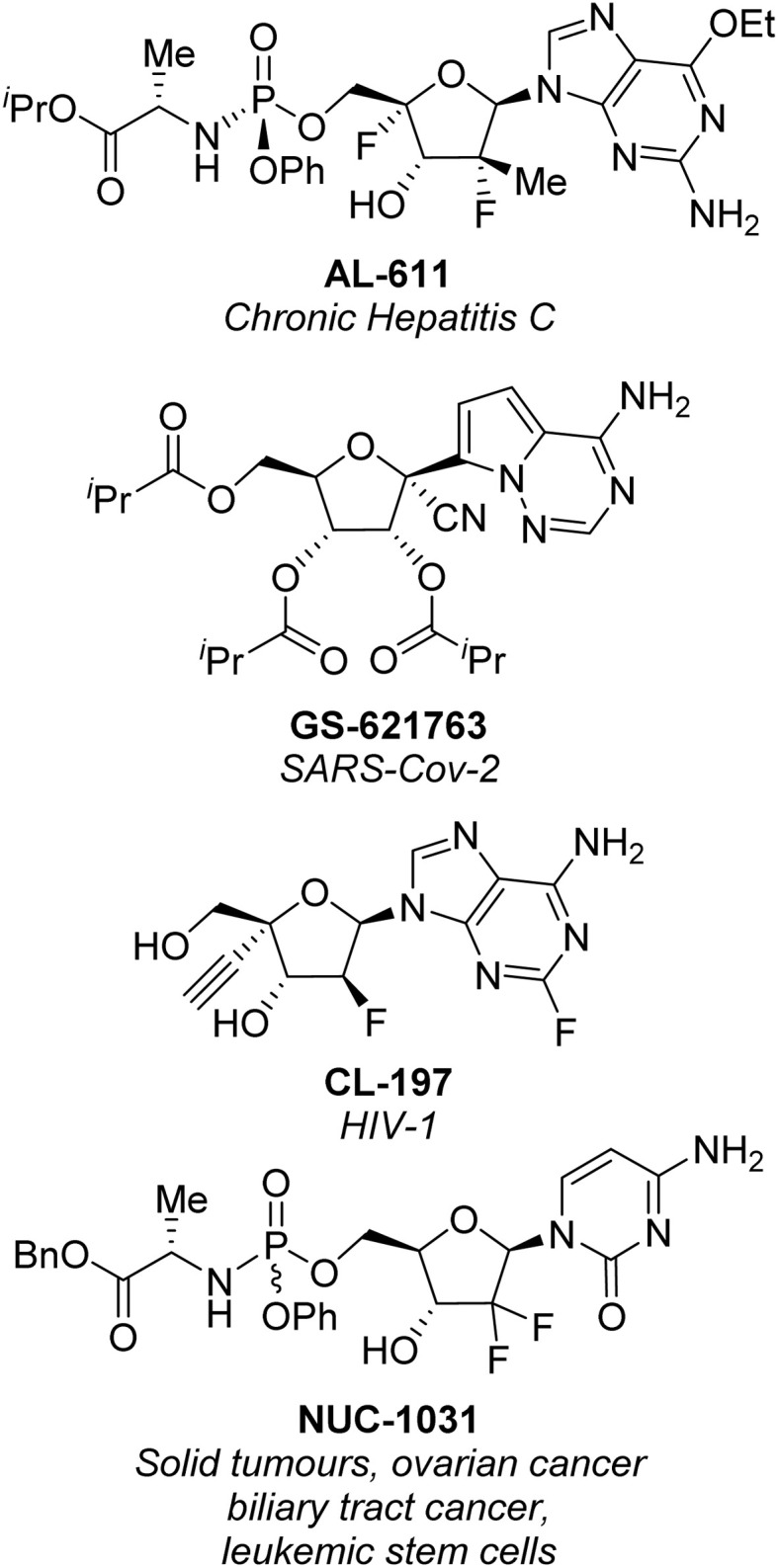
Representative NAs AL-611,^7^ GS-621763,^8^ CL-197,^9^ and NUC-1031,^10^ which have involved new synthetic developments for their preparation over the last 5 years.

Nucleoside analogues (NAs) are a class of molecules which feature prominently across the biotechnology and pharmaceutical sectors.^1^ NAs retain the basic structure of naturally occurring nucleosides, but contain strategically important modifications to the sugar moiety as well as the nucleobase (Fig. 1).^2^ These modifications are essential to enhance efficacy as an antiviral or anti-cancer agent,^3,4^ or to reduce immunogenicity when these analogues are incorporated into RNA vaccines.^5,6^

The demand for step efficient synthetic approaches which incorporate modifications to the sugar moiety in a stereoselective fashion or to prepare NAs comprising non-canonical heterocycles as the nucleobase surrogate has rapidly increased over the last 5 years.^11–13^ This has been spurred on by the recent outbreak of SARS-Cov-2,^14^ resulting in an increase in research activity to prepare antiviral NAs and mRNA vaccines.^15,16^ Combined with the ever-present need for novel modifications for the development of therapeutic oligonucleotides,^17^ there has been a resurgence in both synthetic and biocatalytic methodologies to access novel nucleoside chemical space.^13,18,19^ The purpose of this perspective article is to highlight the key synthetic advances in both of these areas over the last five years, and suggest future opportunities for researchers to explore integrating these approaches for the scalable development of next-generation NA scaffolds.

## General synthetic strategies for nucleoside analogue synthesis

**Fig. 2:**
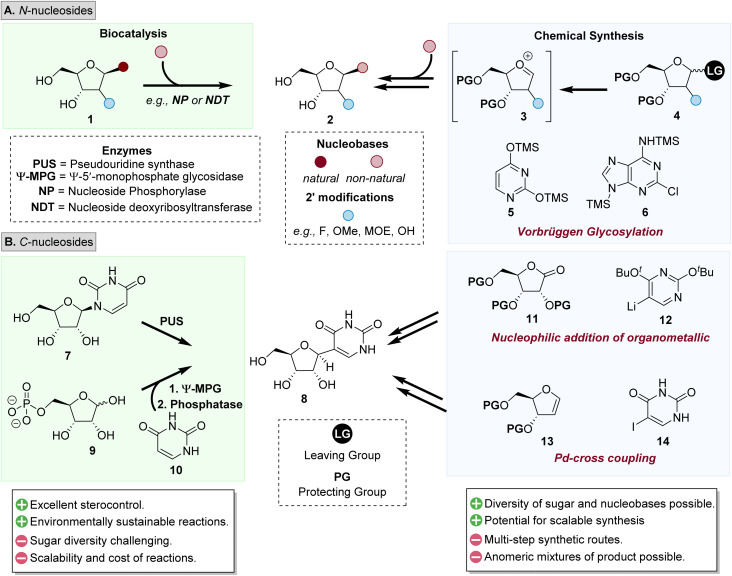
Overview of some of the prominent synthetic approaches to access (A) *N*-, and (B) *C*-nucleosides.

Traditional approaches used to prepare *N*-nucleoside analogues have predominantly relied on the formation of the glycosidic linkage using sugar analogues bearing a leaving group (LG) at the C1′ and a corresponding nucleobase analogue acting as the corresponding nucleophile (Fig. 2A).^18,20,21^ From a biocatalytic perspective, ‘base swapping’ approaches provide efficient access to *N*-nucleoside analogues (2) where natural nucleosides (*e.g.*, 1) act as electrophilic ‘sugar donors’ which undergo nucleophilic attack by a corresponding nucleobase analogue. A cognate chemical synthesis approach is the Vorbrüggen glycosylation route^22,23^ where C1′-halosugars (*e.g.*, 4) are precursors for the *in situ* formation of an oxocarbenium species 3, which are subsequently *N*-glycosylated by silylated nucleobases (*e.g.*, 5 and 6) to form the desired β-nucleoside product 2.

In contrast to the variety of preparative methods explored for *N*-nucleoside synthesis, general preparative routes to access *C*-nucleosides have been more challenging to establish.^21,24^ Recent innovation in biocatalytic routes, such as the isomerisation of uridine 7 catalysed by pseudouridine synthase (PUS),^25^ or the use of ψ-monophosphate glycosidase (ψ-MG)^26^ to form pseudouridine 8*via* the glycosylation of hemiacetal substrates (9) using nucleobase 10 offer intriguing potential to further develop engineered enzymes to access *C*-nucleoside analogues.^27,28^ Chemical synthesis methods have predominantly focused on nucleophilic attack of an organometallic species (*e.g.*, 12) with electrophilic lactones (*e.g.*, 11), radical-based cross-couplings,^29^ or Pd-catalysed cross-coupling strategies,^30^ with a representative example being the reaction between glycal 13 and a corresponding halonucleobase, such as 14.^31^

One of the most enduring challenges in NA synthesis is stereoselective control of these reactions to afford the more desirable β-anomer (*e.g.*, 2).^12,13,32^ Although the use of protecting groups and the need for multi-step synthetic transformations are required, industry has developed scalable strategies for specific nucleoside targets. Whereas biocatalytic efforts have the potential to reduce the synthetic step count, this does require extensive enzyme engineering to broaden the substrate scope in the sugar moieties that can be accessed on scale.^33,34^ Therefore, there exists tremendous opportunity for the development of integrated strategies to harness the advantages of biocatalysis and chemical synthesis, and consequently establish synthetic platforms to access these high value substrates.^18,35–37^

## Exemplar synthetic strategies to access nucleoside analogues incorporating sugar modifications

### C1′-modified nucleoside analogues

Modifications to the sugar C1′ position has emerged as a major site for the development of NAs possessing antiviral and antibacterial activity.^14^ This is due to the evolutionary divergence of viral polymerases (*e.g.*, RNA-dependent RNA polymerases) compared to mammalian polymerases.^38^ The installation of small modifications at this position has been a highly effective medicinal chemistry strategy for the development of remdesivir (22), which possess broad spectrum anti-viral activity against hepatitis C virus (HCV), yellow fever virus (YFV), dengue-2 virus (DENV-2), influenza A (INVA), Ebola virus (EBOV) and severe acute respiratory syndrome coronavirus (SARS-CoV-2).^39–41^ With regards to 22 the installation of the C1′ is essential for its antiviral activity, which involves a delayed chain termination event.^42–44^

**Fig. 3:**
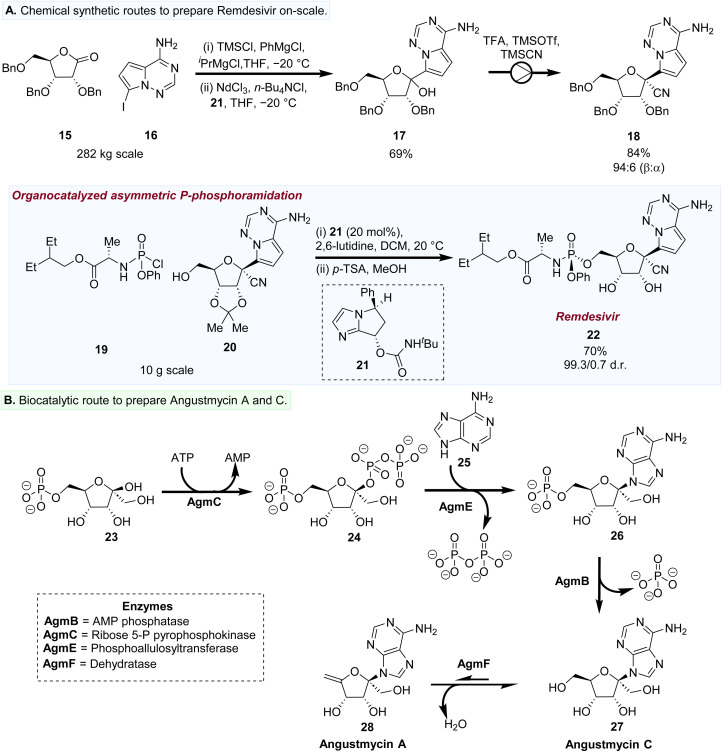
(A) Chemical synthetic routes to prepare remdesivir (22) using a combination of batch and flow-based processes. (B) Biosynthetic routes used to form angustmycin A (28) and C (27).

As a consequence of its broad ranging biological activity, a variety of synthetic efforts have been explored for the scalable preparation of 22 (Fig. 3A).^45^ One prominent example is the synthesis of 22 developed by co-workers at Gilead.^46^ First, a scalable batch-based method of *C*-glycosylation was established using lactone 15 as the sugar donor species. Lithiation of 16 followed by addition to 15 afforded an anomeric mixture of the *C*-nucleoside 17. A critical development was a flow-based cyanation method, affording the desired β-anomer (96 : 4 β : α) 18 in 84% yield.

A marked improvement in the antiviral activity of the remdesivir nucleoside core was observed when the NA was administered as a protide. However, there was a disparity in biological activities of the *R versus S* diastereomers of these protide phosphoramidates,^39,47^ with the *S* diastereomer being far more potent. An organocatalytic approach has recently been developed which formed the desired *S*-diastereomer of 22 in 70% yield and 99.3/0.7 d.r.^48^ Both of these methods showcase the scalability of these chemical synthetic steps to produce a target C1′-modified NA.

Two recent studies have reported the utility of biocatalytic pathways of two NA natural products bearing a C1′-hydroxymethyl group.^49,50^ Angustmycin A and C are biosynthesised by *Streptomyces angustmyceticus* JCM 4053, and exhibit broad range antibacterial and antitumour properties.^51,52^ Yu *et al.* revealed that both of these NA natural products are biosynthesised by five enzymes AgmB, C, E and F (Fig. 3B). First, a pyrophosphokinase (AgmC) catalyses the pyrophosphorylation at the C1′ position of 23 to form 24. The diphosphate 24 at this C1′ position acts as a leaving group for *N*-glycosylation with adenine (25) catalysed by AgmE to form the NA 26. Phosphatase AgmB catalyses phosphoryl cleavage of 26 to afford the free NA 27 (Angustmycin C). Finally, dehydration of the 5′-hydroxymethyl group by a dehydratase (AgmF) afforded angustmycin A (28). At present, there is no structural data on the key enzymes involved in the key steps associated with *N*-glycosylation (AgmE) and dehydration of 27 to form 28. As such, the potential of widening the substrate scope of these enzymes by engineering approaches is potentially an exciting next phase for the development of biocatalytic approaches to prepare C1′-modified NAs (Fig. 3).

### C2′-modified nucleoside analogues

The C2′ position of nucleosides is the most prominent derivatised site on a nucleoside sugar scaffold.^4,19^ From a therapeutic oligonucleotide perspective, modification of the C2′ position improves metabolic stability against nuclease cleavage as well as being a strategic site to tune the pharmacokinetic/pharmacodynamic properties of NAs.^53,54^ The C2′ position also interacts with key amino acid residues in viral polymerases, rendering it a strategic site for antiviral development.^14^ Extensive efforts have been made to expand the chemical space of C2′ modifications in NAs, with the most common synthetic methods focused on either the construction of sugar precursors, followed by installation of a nucleobase *via* a glycosylation step, or *via* nucleophilic attack of a corresponding electrophilic site on an in-tact nucleoside scaffold.^4,41^

**Fig. 4:**
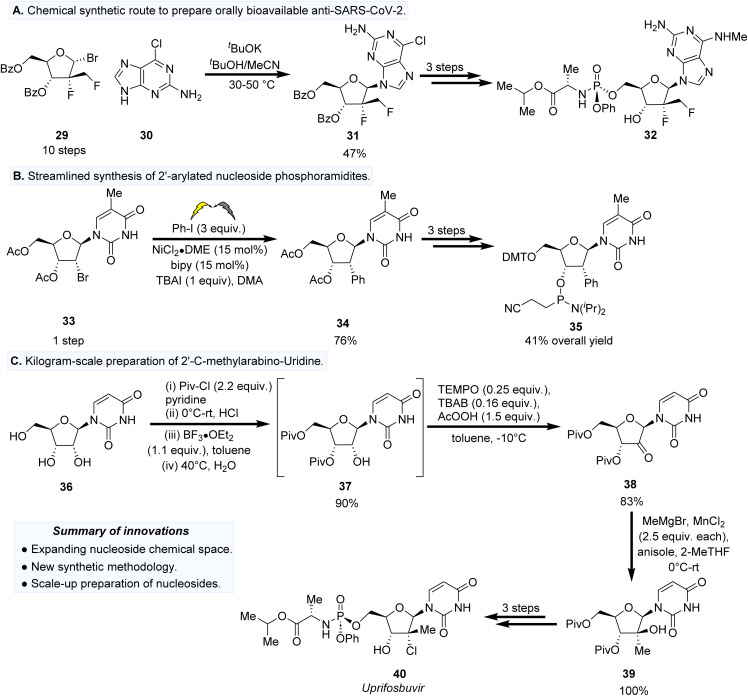
(A) Synthesis of 2′-α-fluoro-2′-β-C-(fluoromethyl) nucleoside phosphoramidate (32) as a lead compound for the treatment of SARS-CoV2 infections. (B) The use of electrochemical methods for the preparation of 2′-arylated nucleoside phosphoramidite (35). (C) Practical kilogram-scale preparation of 39, a key intermediate in the preparation of uprifosbuvir.

The intersection between the development of new synthetic methodology to prepare NAs with the exploration of their biological properties is therefore essential to streamline the trajectory of lead compounds towards clinical applications. We highlight the importance of reconciling both areas, with several exemplars. Liang *et al.* have recently reported a doubly C2′-modified NA exhibits anti-SARS-CoV-2 activity against the 20SF107 strain (EC_50_ = 0.96 ± 0.23 μM) and the Omicron BA.5 variant (EC_50_ = 0.96 ± 0.23 μM) with low cytotoxicity.^55^ The preparation of the ribose donor precursor 29 required a lengthy 10 step synthesis to install the 2′-α-fluoro-2′-β-C-(fluoromethyl) groups as well as the C1′ bromo substituent for subsequent *N*-glycosylation with 30 to form 31 (Fig. 4A). Three additional synthetic steps are required to prepare the phosphoramidite prodrug 32. Whilst the synthetic effort to prepare analogues such as 32 is extensive, this provides inspiration for synthetic chemists to develop new synthetic methodology that accesses these scaffolds in a more step-efficient and environmentally sustainable fashion.

Facile Ni-catalysed cross coupling methodology has recently been reported by Wang *et al.* to prepare C2′-arylated nucleosides (Fig. 4B).^56^ Using readily accessible C2′-brominated nucleoside precursors such as 33 affords the C2′-arylated product 34 using iodobenzene as the coupling partner. Key to this development is the use of electrochemistry to tune the redox potential within the reaction cell, affording a series of C2′-arylated nucleoside products in a single preparative step. The step efficiency of this approach was demonstrated by the preparation of phosphoramidite 35 in a total of five preparative steps in an overall yield of 41%. Incorporation of the 35 into a representative oligonucleotide scaffold by solid phase synthesis exhibited resistance to exonuclease I cleavage, thus providing potential for the further investigation of incorporating these building blocks at strategic sites within a therapeutic oligonucleotide scaffold.

A scalable process to prepare a doubly C2′-modified nucleoside 39 has been reported.^57^ Critical to the synthesis of intermediate 39, Chung *et al.* optimised protection conditions to prepare C3′/C5′ pivaloyl-protected 37 from uridine (36). Oxidation of the C2′-hydroxyl afforded 38 in 83% yield, followed by C2′-methylation to prepare 39. The scalability of these synthetic steps to 39 were conducted on kilogram scale, which streamlines routes towards the antiviral nucleoside analogue uprifosbuvir (40).^58,59^

**Fig. 5:**
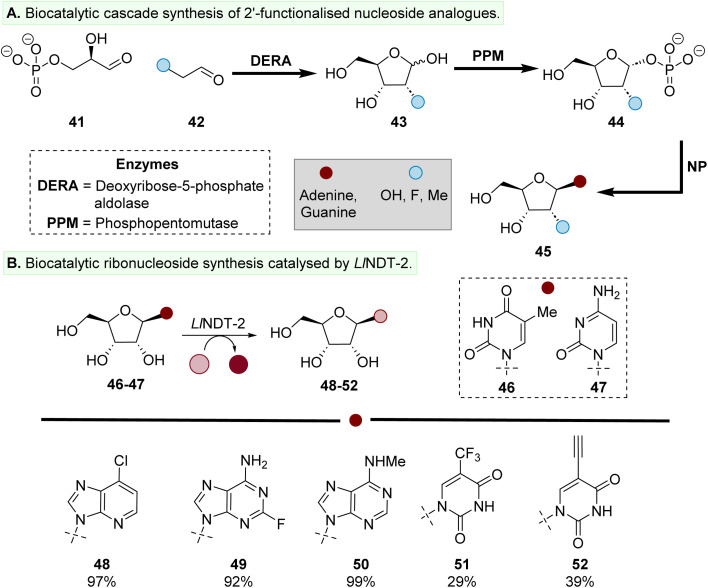
Preparation of ribonucleosides and 2′-modified analogues using a (A) three enzyme reaction cascade and (B) enzymatic transglycosylation catalysed by *Ll*NDT-2.

Exploiting enzymes associated with nucleoside salvage pathways have enabled the preparation of NAs either with isolated enzymes^60^ or as part of enzymatic cascades.^36^ Willmott *et al.* has recently described an elegant three enzyme reaction cascade to prepare C2′-modified NAs using acyclic precursors.^61^ Key to this development was the identification of engineered variants of *Escherichia coli* deoxyribose-5-phosphate aldolase (*Ec*DERA) to accept a variety of donor aldehyde substrates (42), which then catalyses the reaction with 41 to form 43 (Fig. 5A). The double mutant *Ec*DERA-L20A/F76A accepted a variety of aldehydes (42), including C2′-modifications found within therapeutic oligonucleotides, such as methoxy (–OMe), fluoro and methoxymethyl (MOE) groups. The enhanced substrate promiscuity of *Ec*DERA-L20A/F76A was then applied to prepare a variety of therapeutically relevant building blocks *via* phosphorylation of the C1′-OH by phosphopentomutase (PPM) to form 44, followed by glycosylation catalysed by NP to form 45. This three-enzyme cascade was then ameliorated to include enzymatic synthesis of acyclic precursors 41 and 42. This strategy enables access to sugar substrates not readily accessible directly from natural sugars or *via* other chemical synthetic routes.

One drawback with the use of NPs is the formation of nucleosides such as 45 is in equilibrium with precursor 44. The equilibrium favours 44,^12^ with various methods explored to bias the equilibrium towards the formation of the desired nucleoside product, 45.^62–64^ Nucleoside transglycosylases, such type II nucleoside 2′-deoxyribosyltransferase from *Lactobacillus leichmannii* (*Ll*NDT-2), offer an alternative biocatalytic approach to NPs (Fig. 5B).^65,66^ Transglycosylases such as *Ll*NDT-2 catalyse *N*-glycosylation *via* covalent catalysis,^67,68^ thereby avoiding undesirable equilibrium reactions which occur with NPs. Our group has explored the substrate scope and scalability of *Ll*NDT-2 to catalyse the formation of nucleoside analogues (*e.g.*, 48–52) from nucleoside precursors (*e.g.*, 46–47, Fig. 5B).^69,70^ The wildtype *Ll*NDT-2 enzyme readily accepted a variety of modifications to the nucleobase as well as C2′-modifications, including hydroxyl and fluoro substituents in the *ribo* and *arabino* configuration. An alternative enzymatic strategy to access 2′-ribonucleosides has been reported by Genz *et al.* Catalysed by a pyrimidine nucleoside 2′-hydroxylase (PDN2′H), this approach offers the potential to use 2′-deoxyribonucleoside substrates as feedstocks for stereospecific hydroxylation.^71^

### C3′-modified nucleoside analogues

Early iterations of C3′-modified NAs focused on the replacement of the C3′-OH with chain terminating groups, with many examples displaying antiviral and antitumour properties.^72^ A recent iteration in the development of antitumour nucleosides was the identification of 53 as an inhibitor of the ecto-5′-nucleotidase, CD73. CD73 is a receptor which catalyses the degradation of ATP to adenosine.^73^ CD73 is overexpressed in a variety of cancers and plays a crucial role in facilitating tumour growth and metastasis, rendering this receptor as a desirable target for chemotherapeutic intervention.^73^ Applying a structure-guided approach, Li *et al.* identified that modifications to the C3′-position and isosteric groups to mimic the phosphodiester groups were critical for potent inhibition of CD73. Further structure-activity profiling of the C5′-position identified lead compound 53, which exhibits potent inhibition of human CD73 (IC_50_ = 0.17 nM). The authors proposed that a favourable π-interaction between the C-3′-β-alkynyl moiety and the purine ring underpins this increase in potency.
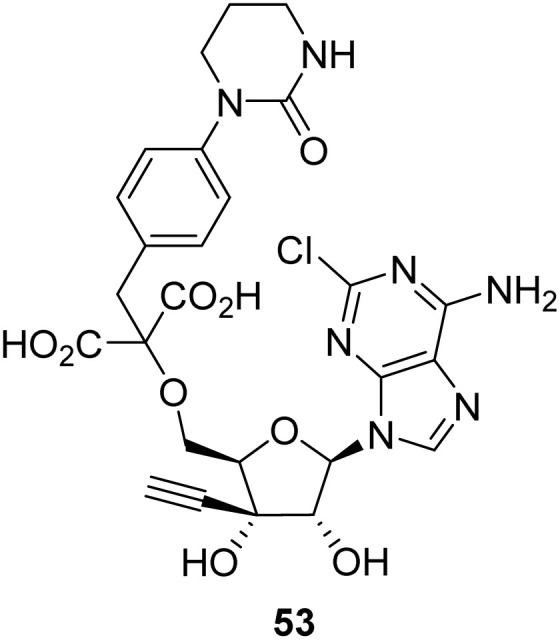


### C4′-modified and bicyclic nucleoside analogues

**Fig. 6:**
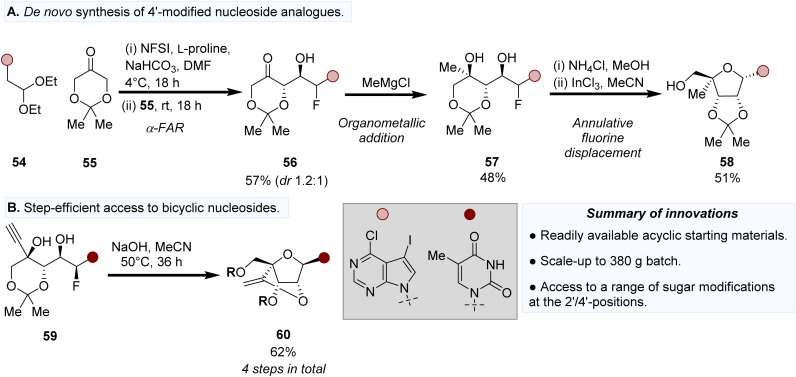
*De novo* synthesis of nucleosides starting from acyclic precursors. (A) Preparation of 4′-modified and (B) bicyclic nucleoside analogues.

Strategic installation of modifications in the C4′-position has been desirable for the formation of locked nucleic acid building blocks for therapeutic oligonucleotide applications,^74,75^ as well as their ability to influence sugar puckering.^76^ Whilst traditional routes have involved linear and often bespoke synthetic routes to first prepare the ribose sugar donor followed by a final glycosylation step,^77^ a recent innovation by the Britton group has developed a *de novo* synthesis of C4′-modified NAs from acyclic precursors. Much akin to the corresponding biocatalytic cascade routes mentioned earlier (Fig. 5A),^36,61^ Meanwell *et al.* developed a cognate synthetic approach involving firstly a l-proline-catalysed α-fluorination and aldol reaction (α-FAR) between 54 and 55 to form 56 as an epimeric mixture (Fig. 6A).^78^ Organometallic addition to the ketone in 56 afforded 57, which then underwent a Lewis-acid catalysed annulative fluorine displacement (AFD) reaction to afford C4′-modified NAs such as 58. This chemical synthesis-based approach is unique amongst the stable of other approaches as it provides in-built flexibility to access a variety of different modification types in a minimal number of steps. For example, step-efficient access to 56 derived from an α-FAR reaction enables installation of a variety of modifications in the C4′-position. Grignard addition to 56 by ethynylmagnesium bromide, afforded 59, which can then undergo an AFD and subsequent cyclisation to form a bicyclic analogue (60, Fig. 6B). Reduction of 56 to the corresponding diol (57) followed by AFD enables step-efficient access to ribonucleoside analogues (58), thus providing further opportunities for scaffold diversification.^79,80^

### Thionucleoside analogues

**Fig. 7:**
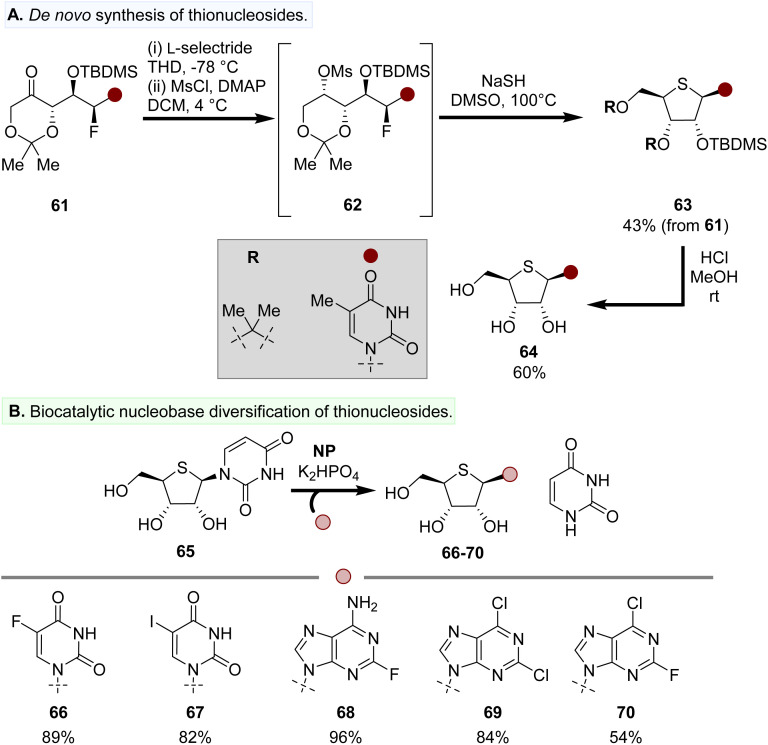
Preparation of thionucleosides by (A) *de novo* synthesis and (B) a biocatalytic base swapping approach.

An extension to Britton's *de novo* nucleoside synthesis was recently demonstrated for the preparation of thionucleosides. Reduction of the acyclic precursor 61 and mesylation afforded 62*in situ*, which was telescoped through to the formation of thionucleoside 63 by the addition of NaSH and heated to 100 °C (Fig. 7A).^81^ Acetal deprotection of 63 afforded free thionucleoside analogues (64).

An enzymatic ‘base-swapping’ approach using a central thionucleoside precursor such as 65 as the thiosugar ‘donor’ has been reported by Westarp *et al.*, providing a divergent synthetic route to prepare thionucleosides outfitted with a variety of different nucleobases from a single thionucleoside precursor (Fig. 7B).^82^ NPs were used as the biocatalyst, which in the presence of both purine and pyrimidine nucleobases afforded thionucleoside products 66–70.

## Summary, outlook and future opportunities

The realisation of the clinical potential of therapeutic oligonucleotides^83–85^ in combination with the identification of remdesivir and molnupiravir for the treatment of SARS-CoV2 infections^86^ and mRNA vaccines has reinvigorated the need to develop methodologies for the preparation of NAs. Whilst traditional synthetic routes have focused on either glycosylation or building up nucleobase analogues with appropriately derivatised sugar moieties,^4,41^ the last five years has witnessed innovative methods to build up sugar moieties from acyclic precursors.^79^ Prominent in the field is an increase in the adoption of biocatalytic cascade methods for this purpose.^18,61,82^ These routes are especially pertinent for the incorporation of NAs into mRNA vaccines or cyclic nucleotides which require subsequent phosphorylation steps of the 5′-OH for triphosphate synthesis or monophosphorylation of capped substrates.^87–89^ Protein engineering in this respect provides untold opportunities to expand the substrate scope of a wide variety of enzymes associated with various synthetic transformations, ranging from sugar synthesis through to glycosylation.^61^

Table 1 summarises the current state-of-the-art in accessing modifications to the nucleoside scaffold using biocatalytic and chemical synthesis methods, and highlights potential opportunities ripe for exploration. Over the last 5 years, there has been an impressive level of innovation in the application of biocatalytic routes to prepare *N*-nucleoside analogues with defined modifications on the C2′ and C4′ sugar scaffold.^20,36,61,69,90,91^ However, the lack of equivalent development of biocatalytic routes to assist in the preparation of NAs with modifications at the C1′ and C3′ positions offers opportunities for further innovation. Similarly, the preparation of *C*-nucleoside analogues have predominantly focused on target-driven methodologies rather than establishing a generalised synthetic platform. The development of generalised biocatalytic^28,92^ as well as chemical synthetic routes^24^ to prepare a wider scope of *C*-nucleosides offers opportunities to develop NA libraries with enhanced metabolic stabilities and bioactivities relative to their *N*-nucleoside cognates.

**Table 1:** Summary of existing methods to modify nucleoside scaffolds, and future opportunities for the development of new synthetic methodologies

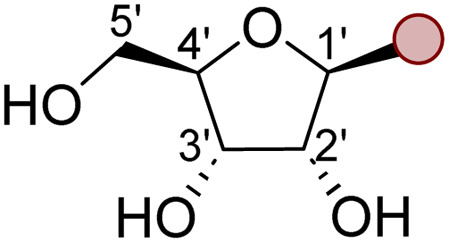			
Modification	Predominant method of synthesis 2020–2025	Current limitations	Opportunities
*C*-Nucleoside	Chemical	Lack of modular synthetic routes	• Modular synthetic methods
			• Explore biocatalytic alternatives
*N*-Nucleoside	Biocatalytic	Substrate scope of sugar analogues	Enhance biocatalytic substrate scope
C1′	Chemical	Lack of modular synthetic routes	Enhance substrate score of emerging biocatalytic routes
C2′	Biocatalytic & chemical	• Step-efficient routes to expand chemical space	Enhance substrate score of emerging biocatalytic routes
		• Biocatalytic substrate scope	
C3′	Chemical	Lack of modular synthetic routes	• Modular synthetic methods
			• Explore biocatalytic alternatives
C4′	Biocatalytic & chemical	Substrate scope of sugar analogues	Diversification of sugar scaffold

Despite recent innovations in both chemical synthetic methodology and in the field of biocatalysis, there is ample room for closer integration of these approaches. Limitations of substrate scope with existing biocatalytic methods offers opportunities to develop sustainable synthetic methods by, for example, preparing key intermediates which feed into biocatalytic cascades.^93^ In addition, diversifying chemical space of for example, the nucleobase,^94^ or novel sugar substrates provides inspiration for the synthetic chemist to develop new methods. Flow-based approaches are amenable to both chemical synthesis and biocatalysis,^95,96^ which when combined with data analytics and machine learning platforms,^97–99^ offers an auxiliary level of innovation for the synthesis of these high value products.

## Abbreviations

*Ll*NDT-2: Type II nucleoside 2′-deoxyribosyltransferase from *Lactobacillus leichmannii*
NP: Nucleoside phosphorylase

## Author contributions

The manuscript was written through contributions of all authors. All authors have given approval to the final version of the manuscript.

## Conflicts of interest

There are no conflicts of interest to declare.

## Data Availability

No primary research results, software or code have been included and no new data were generated or analysed as part of this review.
